# Live Podcasting as an Educational Intervention in Dentomaxillofacial Radiology: Controlled Cohort Study

**DOI:** 10.2196/77980

**Published:** 2026-01-05

**Authors:** Anna-Lena Hillebrecht, Daniel Fritzsche, Thamar Voss, Anne Kruse, Andreas Keßler, Kirstin Vach, Markus Jörg Altenburger, Rainer Schmelzeisen, Wiebke Semper-Hogg

**Affiliations:** 1 Department of Prosthetic Dentistry, Center for Dental Medicine, Faculty of Medicine, Medical Center, University of Freiburg, Hugstetter Strasse 55, Freiburg, 79106, Germany, 49 76127048440; 2 Department of Operative Dentistry and Periodontology, Center for Dental Medicine, Faculty of Medicine, Medical Center, University of Freiburg, Freiburg, Germany; 3 Department of Educational Science, University of Freiburg, Freiburg, Germany; 4 Department of Conservative Dentistry and Periodontology, University Hospital, Ludwig-Maximilians University of Munich, Munich, Germany; 5 Institute of Medical Biometry and Statistics, Medical Center, University of Freiburg, Freiburg, Germany; 6 Department of Orthodontics, Center for Dental Medicine, Medical Center, Faculty of Medicine, University of Freiburg, Freiburg, Germany; 7 Department of Oral and Maxillofacial Surgery, Center for Dental Medicine, Faculty of Medicine, Medical Center, University of Freiburg, Freiburg, Germany

**Keywords:** case-based learning, clinical reasoning, dental education, digital learning, health professions education, interdisciplinary teaching, podcast, student engagement, synchronous learning

## Abstract

**Background:**

Podcasts are increasingly used in health professions education; however, most formats are asynchronous and noninteractive. Didactically grounded, synchronous implementations in dental curricula are scarce.

**Objective:**

This study aims to design, implement, and evaluate a synchronous, case-based live podcast (LP) as a didactic teaching format in dentomaxillofacial radiology.

**Methods:**

In a controlled cohort study with 2 third-year cohorts (N=41), the intervention group (IG; n=21, 51%) received weekly case-based LP sessions in addition to standard teaching, while the control group (CG; n=20, 49%) received standard teaching only. Acceptability was evaluated 6 months postcourse using the 27-item student evaluation questionnaire and open-text responses. Knowledge was assessed immediately after the course with a 21-item radiology knowledge test, and after 6 months, with a 15-item interdisciplinary clinical application test.

**Results:**

The primary outcome was student-reported acceptability of the LP format. It was rated highly by students in the Student Evaluation Questionnaire (mean out of 10: structure 9.76, interactivity 9.62, interdisciplinary relevance 9.55). Qualitative feedback was assessed highlighting motivation, authenticity, and discussion quality. In the radiology knowledge test, no group differences were observed (IG: n=21, 51% vs CG: n=20, 49%; *P*=.37). In the interdisciplinary clinical application test, the IG outperformed the CG in restorative dentistry (median 5, IQR 4-5 vs median 4, IQR 3-5; *P*=.02; *r*=0.38) and in item-level analysis (15/21, 71% vs 40%; *P*=.04; *φ*=0.64).

**Conclusions:**

The LP format represents a feasible, scalable, and low-threshold approach to fostering clinical reasoning in dental curricula, particularly at the transition to clinical training. While radiology-specific theoretical competencies did not differ between the groups, students consistently rated the LP as more engaging and motivating compared to standard lectures.

## Introduction

### Overview

In the landscape of health professions education, digital audio formats, particularly podcasts, have gained substantial momentum [1-5]. Their flexibility, accessibility, and conversational tone have made them an increasingly attractive medium for knowledge dissemination and learner engagement [4,6]. While podcasts are now widely accepted as a complementary teaching method in undergraduate medical education, their use remains predominantly asynchronous and prerecorded, with limited interactivity and integration into curricular strategies [1]. Despite their potential, podcasts have rarely been explored as intentionally designed didactic tools grounded in contemporary educational theory. While the demand for flexible and digital teaching formats in dental education continues to rise, there is a striking lack of pedagogically grounded concepts specifically addressing the use of podcasts within the dental curriculum [5,7]. To better understand students’ needs in modern education, a preliminary needs assessment was conducted among undergraduate dental students at the University of Freiburg. The results indicated that while students appreciated classical media such as lectures and textbooks for foundational learning, they strongly favored digital formats for more advanced clinical content. Podcasts, although not yet widely used for learning, were perceived as a promising format for flexible, self-directed studies. These findings highlighted a clear interest in diverse, interactive, and clinically relevant digital learning formats [8].

### Rationale and Objectives

Recent initiatives such as the student-led technology committee highlight the growing recognition of students as active stakeholders in shaping digital education, demonstrating how learner involvement can drive innovation and relevance in curricular technologies [9]. At the same time, in dentistry, clinical reasoning (CR) is gaining increasing importance, particularly as dental treatment needs are rising among vulnerable populations, including older adults and patients with complex medical conditions [10,11]. CR is particularly evident in dentomaxillofacial radiology (DMFR), where diagnostic decisions based on radiographic interpretation significantly influence treatment planning. CR in this context includes not only the technical analysis of imaging data but also interdisciplinary judgment, risk assessment, and patient-centered decision-making. CR refers to the cognitive process by which health care professionals collect, interpret, and synthesize patient information to make informed diagnostic and therapeutic decisions. CR remains a central yet pedagogically challenging competence in medical and dental education. Developing CR requires authentic, situated learning experiences that bridge disciplines and promote critical discussion, an area in which traditional lecture-based formats often fall short.

In recent years, traditional lecture-based formats have increasingly lost their appeal among students, raising the question of whether innovative and more interactive approaches can reengage learners and potentially revitalize attendance in academic teaching. This study introduces and evaluates the live podcast (LP) as an innovative digital teaching format that combines the accessibility of podcasting with the didactic strengths of interactive, case-based learning. To our knowledge, this is the first systematic implementation of an LP format within the dental curriculum. The LP was not conceived as a mere content delivery tool but as a didactically grounded educational intervention designed to foster clinical reasoning, interdisciplinary thinking, and student engagement through authentic expert dialogue. The objective was to introduce a didactically designed LP format within undergraduate DMFR and evaluate its feasibility and student-reported acceptability and to explore potential effects on theoretical and interdisciplinary knowledge compared with standard teaching.

The LP format was hypothesized to be feasible, well accepted by students, and to improve knowledge scores in domains requiring case-based reasoning compared with standard instruction.

## Methods

This study primarily presents and evaluates the LP as a novel teaching format in DMFR education, focusing on its instructional design, implementation, and initial effectiveness.

### Curricular Context

Undergraduate dental education in Germany is structured as a 5-year program, consisting of a 2-year preclinical phase (basic sciences such as anatomy, physiology, biochemistry, and histology, along with introductory dental subjects), followed by a 3-year clinical phase. The curriculum is nationally regulated and guided by the National Competence-Based Learning Objectives Catalogue in Dentistry. DMFR is introduced at the beginning of the clinical training (fifth semester) in close connection with basic dental instruction and is subsequently applied throughout the clinical phase. The course includes radiographic techniques, diagnostic interpretation, radiation protection, and ethical considerations, integrating theoretical instruction with practical training (intraoral radiographs, including periapical radiographs, bitewing radiographs, and occlusal radiographs; panoramic radiographs such as orthopantomogram; and cone-beam computed tomography). The LP sessions were designed based on National Competence-Based Learning Objectives Catalogue in Dentistry objectives, particularly Z 21 (clinical diagnostics, radiographic imaging, and radiation protection) and Z 5 (dentist as medical expert), thereby ensuring consistency with national competency standards.

### Didactic and Conceptual Design

The LP was developed as a synchronous, case-based learning format and implemented as a mandatory component of the DMFR course for third-year undergraduate dental students at the University of Freiburg. Each weekly 45-minute session followed a structured design: anonymized real-life cases were discussed in moderated dialogue between a dental radiologist and a specialist from a complementary dental or medical discipline (eg, pediatric dentistry, prosthodontics, oral surgery, and radiology). Discussions focused on radiographic diagnosis, differential diagnoses, and interdisciplinary treatment planning. All episodes were designed with explicit learning goals that emphasized diagnostic competence and clinical reasoning, in line with the interdisciplinary nature of DMFR (Tables1  2).

**Table 1.** Curriculum framework for the live podcast: episodes, topics, disciplines, imaging modalities (periapical radiographs [PA], bitewing radiographs [BW], occlusal radiographs [OC], panoramic radiographs such as orthopantomogram [OPG], and cone-beam computed tomography [CBCT]), and radiographic diagnoses.

Episode	Topic	Discipline	Imaging modalities in this episode	Radiographic diagnoses
1	Pediatric dentistry	Pediatric dentistry	PA; BW; OPG	Approximal caries and apical periodontitis
2	Endodontology	Endodontics	PA; CBCT for complex cases	Irreversible pulpitis and apical periodontitis
3	Caries diagnostics	Restorative dentistry	BW; PA	Bitewing caries; secondary caries versus artifacts
4	General prosthodontic planning	Prosthodontics	OPG; PA	Tooth loss, apical periodontitis, bone loss, and TMJ^a^ findings
5	Special care dentistry	Special care dentistry	OPG; PA	Tooth loss, caries, bone loss, and radiation protection issues
6	General radiology	Oral and maxillofacial radiology	PA; BW; OPG; CBCT; MRI^b^	Modality characteristics (intraoral, BW, CBCT, and MRI)
7	History of dentistry	History of medicine or dentistry	Historical imaging methods	Early radiographs and OPG
8	Artificial intelligence in dentistry	Informatics; oral radiology	PA; OPG; CBCT	AI^c^-assisted detection of caries and periodontal bone loss
9	Orthodontics	Orthodontics	OPG; lateral cephalogram; PA; OC	Tooth development; retained teeth
10	Periodontology	Periodontology	PA; BW; OPG	Horizontal and vertical bone loss
11	Surgical removal of wisdom teeth	Oral and maxillofacial surgery	OPG; CBCT for complex cases	Impactions, cysts, and nerve injury risk

a TMJ: temporomandibular joint.

b MRI: magnetic resonance imaging.

c AI: artificial intelligence.

**Table 2.** Curriculum framework for the live podcast: episodes, topics, covered domains, interdisciplinary aspects, and learning goals.

Episode	Topic	Covered domains	Interdisciplinary aspects	Learning goals
1	Pediatric dentistry	Imaging and diagnostics in primary and mixed dentition, trauma management, prevention, and developmental diagnostics	Pediatrics, and oral and maxillofacial surgery (fracture management)	Apply pediatric-specific diagnostic strategies, and select imaging in pediatric trauma and explain informed consent in minors
2	Endodontology	Pain diagnostics, pulp vitality assessment, and decision-making in endodontic emergencies	Oral and maxillofacial surgery and restorative dentistry	Strengthen case-based reasoning and interpret findings in irreversible pulpitis and differentiate periapical pathologies
3	Caries diagnostics	Primary and secondary caries, diagnostics, and prevention	Prevention, pediatrics, prosthodontics, and endodontics	Enhance diagnostic accuracy and apply imaging in caries diagnostics and distinguish true lesions from imaging artifacts
4	General prosthodontic planning	Prosthetic concepts, treatment planning, and radiographic considerations	Geriatrics, orthodontics, oral and maxillofacial surgery, and restorative dentistry	Integrate diagnostics into treatment planning, and use OPG^a^ in planning and develop interdisciplinary prosthodontic strategies
5	Special care dentistry	Prosthetic care for people with disabilities, barriers to care, and ethics	Medicine, nursing, and ethics (focus on vulnerable and medically complex patients)	Link ethics with radiology, and apply radiation protection in vulnerable groups and critically reflect on imaging indications
6	General radiology	Physics; ALARA^b^ principle; imaging modalities	Cross-disciplinary relevance of imaging decisions	Build safety and modality competence, and apply ALARA and differentiate indications for imaging modalities
7	History of dentistry	Evolution of diagnostics and imaging	History of medicine	Promote scientific reflection, and describe historical developments in dental diagnostics and radiology
8	Artificial intelligence in dentistry	Digital diagnostics and decision support	Ethics and data science	Foster critical, future-oriented thinking; evaluate benefits and limitations of AI^c^-assisted diagnostics
9	Orthodontics	Malocclusions and growth diagnostics	Pediatrics and prosthodontics	Relate radiology to growth analysis, and explain OPG or CBCT^d^ and model analysis in orthodontics and assess impacted teeth
10	Periodontology	Periodontal diagnostics, disease progression, and treatment planning	Prosthodontics and implantology	Strengthen diagnostic integration, and identify periodontal bone loss radiographically and link findings to implant and prosthodontic planning
11	Surgical removal of wisdom teeth	Indications, surgical techniques, and preoperative planning	Radiology, anesthesiology, and prosthodontics	Link diagnostics with surgery, and select OPG versus CBCT appropriately and assess surgical risks radiographically

a OPG: orthopantomogram.

b ALARA: As Low As Reasonably Achievable.

c AI: artificial intelligence.

d CBCT: cone-beam computed tomography.

### Technical Setup

Sessions were held in a lecture hall with professional audiovisual infrastructure. At least 2 microphones transmitted the voices of the podcasters via the hall sound system, and an additional microphone was available for student interaction. A projector displayed radiographs and clinical photographs. Each session was recorded live with sound levels adjusted by a technician. Recordings were postprocessed in Adobe Audition (version 23.11; Adobe Inc) and uploaded via Panopto (version 14.0; Panopto Inc) to the official university learning platform, ensuring high audio quality and asynchronous access for enrolled students (Multimedia Appendix 1).

### Participants and Study Design

All third-year dental students enrolled in the mandatory DMFR course at the University of Freiburg were eligible. In total, 41 students participated in 2 consecutive cohorts: a control group (CG; n=20, 49%; with n=12, 60% women and n=8, 40% men) that attended traditional lectures covering DMFR principles, radiographic techniques, and clinical relevance, and an intervention group (IG; n=21, 51%; with n=11, 52% women and n=10, 48% men) that attended the LP sessions. No exclusion criteria were applied because all students were required to complete the course as part of the curriculum. The cohorts received comparable curricular content from the same teaching staff, ensuring baseline comparability of exposure. No pretest was conducted because both groups had identical prior curricular exposure. All students completed the subsequent knowledge assessments. Item-level correctness and point-based domain scores were analyzed across restorative dentistry, oral surgery, and orthodontics. The 3 assessment instruments used in this study were the interdisciplinary clinical application test (ICAT; Multimedia Appendix 2), the radiology knowledge test (RKT; Multimedia Appendix 3), and the Student Evaluation Questionnaire (SEQ; Multimedia Appendix 4).

### Assessment of Student-Reported Acceptability

To determine the primary outcome, student-reported acceptability was evaluated 6 months after the LP using an anonymous questionnaire with 27 rating items (SEQ), on a 10-point unipolar scale (1=I strongly disagree, 10=I strongly agree). The questionnaire covered organizational aspects, atmosphere, interactivity, memorability, long-term benefit, integration of theory and practice, reflective engagement, social learning, and overall evaluation. Selected domains were stratified by gender. An optional open-text field captured qualitative impressions that were analyzed descriptively.

### Assessment of Learning Outcomes

Knowledge served as the secondary outcome and was evaluated at 2 time points to capture both immediate and longer-term learning effects.

Immediately after the course: IG and CG completed a DMFR radiology-specific competence test (RKT), with 21 single-choice items (F1-F21) covering radiation protection, diagnostic imaging, and interpretation.

Six months after the course, IG and CG participated in an interdisciplinary multiple-choice test (ICAT) with 15 case-based items (5 items each for restorative dentistry, oral surgery, and orthodontics), addressing radiographic interpretation, interdisciplinary treatment planning, and clinical application of radiological findings.

### Statistical Analysis

No formal sample size calculation was performed. As a feasibility study embedded in routine teaching, the available cohort size (N=41) defined the sample. This aligns with the exploratory aim to evaluate acceptability and didactic value rather than to demonstrate definitive knowledge effects. Descriptive statistics (mean, SD, median, and IQR) were calculated for all outcomes. Chi-square tests were applied for item-specific correctness, and Wilcoxon rank-sum tests were used for group comparisons of point-based scores and gender differences. The Fisher exact test was applied in the case of single-choice items. Effect sizes (*r*) were reported where appropriate. Given the exploratory design, no adjustment for multiple testing was applied. Statistical analyses were performed in Stata (version 19; StataCorp) with a significance level of *α*=.05.

### Ethical Considerations

The study involved human participants and was conducted in accordance with institutional and national research regulations and the Declaration of Helsinki. The study was reviewed informally by the ethics committee of the University of Freiburg (reference number: 25-1479). As it served internal educational quality assurance without collection of personal or health-related data, formal approval was not required. Informed consent was obtained from all participants during their voluntary enrollment in the course after they had received detailed information about the study purpose, procedures, data use, and their right to withdraw at any time without negative consequences. Data were collected and analyzed anonymously and treated confidentially in accordance with data protection regulations. Participation was voluntary and no financial or other compensation was provided.

## Results

### Overview

A total of 41 third-year dental students participated in the study (IG: n=21, 51%; CG: n=20, 49%). All students completed the RKT immediately after the course, and all participated in the ICAT interdisciplinary follow-up test 6 months later. Student-reported acceptability of the LP format was assessed 6 months postcourse; 21 students (100% of the IG) completed the SEQ.

### Primary Outcome: Student-Reported Acceptability

To determine student-perceived acceptability of the LP format, 21 students completed the SEQ 6 months after the course. Overall, the LP received consistently high ratings across all evaluated dimensions, including atmosphere, organization, perceived usefulness, and interdisciplinary relevance. The mean scores were above 9/10 in nearly all categories, indicating very high acceptance and perceived educational value. No meaningful gender−related differences were observed (Table 3).

**Table 3.** Student Evaluation Questionnaire (SEQ): evaluation of the live podcast in different categories stratified by gender.

Category	Women (n=11; score: 1-10), mean (SD)	Men (n=10; score: 1-10), mean (SD)
Atmosphere	9.91 (0.30)	9.40 (0.97)
Organization	9.82 (0.40)	9.70 (0.48)
Useful teaching method	9.73 (0.65)	9.56 (0.73)
Scope of knowledge	9.18 (1.32)	9.56 (1.01)
Interdisciplinary connections	9.45 (0.82)	9.67 (0.50)
Personal perception	6.64 (1.21)	7.67 (1.94)
Addition to the lecture	8.64 (1.75)	8.78 (1.39)
Interest in topics	9.91 (0.30)	9.30 (0.95)

No meaningful gender-related differences were identified for the total evaluation score (P=0.18) or for any subscale (P=0.32–0.79).

**Figure 1.**
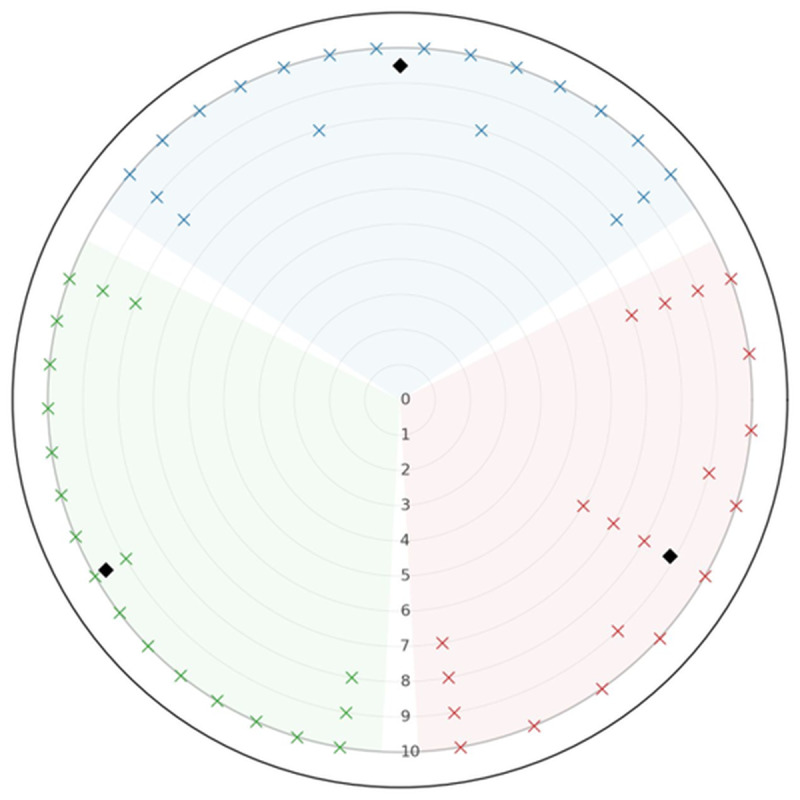
Distribution of participant ratings for the Student Evaluation Questionnaire (SEQ) items. The radial plot visualizes the distribution of all ratings (1-10) for the items “learning from the experiences of others” (blue), “encouraged reflection” (red), and “useful teaching method” (green; each n=20). Each cross represents 1 individual response, while black diamonds (◆) indicate mean values. No ratings were given for scores 1-5. For “learning from the experiences of others,” 20% (n=4) of participants rated 8, 10% (n=2) rated 9, and 70% (n=14) rated 10. For “encouraged reflection,” 5% (n=1) rated 6, 15% (n=3) rated 7, 15% (n=3) rated 8, 20% (n=4) rated 9, and 45% (n=9) rated 10. For “useful teaching method,” 10% (n=2) rated 8, 15% (n=3) rated 9, and 75% (n=15) rated 10. All other rating categories received no responses. A unipolar rating scale from 1 (I strongly disagree) to 10 (I strongly agree) was used, with higher values indicating a more favorable evaluation.

Student ratings for the 3 selected evaluation items as illustrative examples are shown in Figure 1. Overall, the majority of participants provided high ratings, indicating strong agreement across all 3 items. Specifically, for “learning from the experiences of others,” 20% (n=4) of participants rated 8, 10% (n=2) rated 9, and 70% (n=14) rated 10. For “encouraged reflection,” 5% (n=1) rated 6, 15% (n=3) rated 7, 15% (n=3) rated 8, 20% (n=4) rated 9, and 45% (n=9) rated 10. For “useful teaching method,” 10% (n=2) rated 8, 15% (n=3) rated 9, and 75% (n=15) rated 10. All other rating categories received no responses.

### Qualitative Findings

In addition to the quantitative results, students provided written feedback at the end of the course. Selected anonymized comments (translated from German) illustrate how the LP was perceived:

*The podcasts were scheduled at a good time before lunch, which encouraged further discussion of the topics in the cafeteria afterwards. The content was very stimulating.*

*In some sessions, time was even too short, as the discussions were highly engaging. A second microphone for students would sometimes have been helpful.*

*I sincerely appreciate the effort that went into these sessions. It was a great experience to learn the material in such an interesting way through contributions from many different lecturers.*

*Through the podcast I realized how much dentistry has to offer. The podcast, with its many insights into different specialties, gave me renewed motivation and interest in my studies. After four semesters of rote learning, there could not have been a better introduction to the clinical phase.*

*I really enjoyed attending the podcast lectures. They were varied and never felt like dry teaching, yet I learned a great deal. The relaxed atmosphere, such as sitting on the sofa, created an environment that did not feel like studying, although one gained a lot of knowledge.*

These student voices indicate that the LP sessions were experienced as innovative, motivating, and discussion-stimulating, creating a positive learning atmosphere at the transition from preclinical to clinical studies.

### Secondary Outcomes: Knowledge Acquisition

In the ICAT, item-level analysis revealed a significantly higher proportion of correct responses in the IG compared with the CG in restorative dentistry (*P*=.04; φ=0.64). No statistically significant differences were observed in oral surgery (*P*=.85; φ=0.005) or orthodontics (*P*=.14; φ=0.34).

Across all ICAT domains combined, no statistically significant difference in overall performance was observed between groups (*P*=.37; φ=0.49). A distribution-based analysis showed that in the IG, 7 students answered 3 items correctly, 13 students answered 2 items correctly, and 1 student answered 1 item correctly, with no student scoring 0 correct answers. In contrast, in the CG, 4 students answered 3 items correctly, 12 students answered 2 items correctly, 2 students answered 1 item correctly, and 2 students answered no items correctly. This distribution indicates a trend toward higher overall performance in the IG, although the difference did not reach statistical significance (Table 4).

**Table 4.** Interdisciplinary clinical application test (ICAT): point-based domain scores by group (Wilcoxon rank-sum test; *α*=.05)^a^.

Domain	IG^b^ (points), median (IQR)	IG (points), mean (SD)	CG^c^ (points), median (IQR)	CG (points), mean (SD)	Effect size *r*
Restorative dentistry	5 (4-5)	4.62 (0.67)	4 (3-5)	3.75 (1.25)	0.38
Oral surgery	5 (3-5)	3.95 (1.46)	5 (3.5-5)	4.10 (1.37)	0.05
Orthodontics	5 (5-5)	5.00 (0.00)	5 (5-5)	4.60 (1.23)	0.23

a Median, IQR, mean, and SD values are presented for the intervention and control groups. The effect size (*r*) is shown for each domain. Only the comparison in restorative dentistry reached statistical significance (*P*=.02). No significant differences were found for oral surgery or orthodontics.

b IG: intervention group.

c CG: control group.

No statistically significant differences were observed in the RKT between IG and CG for the overall score or any subdomain (all P>.05). The total RKT score was numerically higher in the IG (median 14, IQR 13-15) than in the CG (median 13, IQR 11-14), but this difference did not reach statistical significance (*P*=.17; *r*=0.22), indicating comparable short-term acquisition of radiology-specific theoretical competences in both groups.

In the ICAT, analysis of item-level correctness revealed a significantly higher performance in the IG (75/105, 71%) than in the CG (40/100, 40%) in restorative dentistry (*P*=.04; *φ*=0.64). No significant differences were observed in oral surgery (*P*=.85; *φ*=0.005) or orthodontics (*P*=.14; *φ*=0.34). Overall correctness across all domains was slightly higher in the IG (161/315, 51%) compared with the CG (147/300, 49%), though this difference was not statistically significant (*P*=.37; *φ*=0.49; Table 4).

## Discussion

### Principal Findings

This study demonstrates the development and feasibility of a novel teaching format in undergraduate dental education: the live podcast. To our knowledge, this is the first systematically designed and evaluated synchronous podcast format embedded within a dental curriculum. Unlike traditional podcasts, the LP was conceived as a didactically grounded intervention that combines digital accessibility with case-based, interactive learning. Our results provide initial evidence that LPs are feasible and highly accepted. The consistently high SEQ ratings in structure, clarity, and interactivity suggest that LPs foster an engaging learning environment, particularly at the transition from preclinical to clinical training.

Students in the intervention group performed significantly better in the ICAT in the restorative dentistry domain, which indicates that authentic case discussions supported by expert dialogue may enhance diagnostic reasoning. Although no significant differences were observed across all domains in ICAT, descriptive trends point to positive effects on interdisciplinary understanding. Beyond knowledge outcomes, very high acceptance ratings and open-ended comments show that students perceived the LP as motivating, discussion-enhancing, and well aligned with their learning preferences. In line with contemporary student-voice literature, the LP addressed the cognitive dimension of clinical reasoning and also fostered engagement, reflexivity, and ownership through its interactive and dialogic structure [12]. Goh and Lim [13] argue that dental learning is increasingly conceptualized as situated and relational. The LP exemplifies this by embedding reasoning in authentic, interdisciplinary practice.

The RKT did not reveal significant differences between the 2 groups. For theoretical radiology competencies, both formats appear equally effective. Taken together with the very high acceptance ratings in the SEQ, this suggests that the added value of the LP lies less in measurable short-term knowledge gains and more in providing a motivating, engaging, and student-centered learning environment with an interdisciplinary focus [1].

Evidence from this study suggests that synchronous, audio-based teaching formats such as LPs can act as effective didactic tools in complex, interdisciplinary areas of dental education, including DMFR. By fostering active engagement, real-time dialogue, and case-based reflection, LPs extend the potential of both traditional lectures and asynchronous e-learning. The positive reception aligns with prior literature on the educational benefits of podcasts. Kaplan et al [14] emphasize brevity, narrative structure, and authentic expert dialogue as advantages aligned with adult learning principles and conducive to learner motivation and retention. Consistent with these findings, students in our study reported in the SEQ high levels of didactic clarity, structure, and perceived longer-term benefit, dimensions repeatedly highlighted in podcast-based education [15]. The conversational and interdisciplinary character of the LP mirrors the informal, yet focused tone described by Kaplan et al [14] as central to engagement and professional connectedness. Narrative and storytelling, which support sense-making, empathy, and professional identity formation [16], were intentionally embedded through authentic case discussions. Qualitative feedback indicated that these elements were experienced as engaging and professionally meaningful, resonating with sociocultural learning theory [14,17]. In line with work that identifies podcasts as vehicles for democratizing discourse and fostering reflection [18], live podcasting may strengthen interdisciplinary awareness, reflective reasoning, and inclusive dialogue in dental education.

Core disciplinary knowledge was tested in the RKT immediately after the course to capture short-term acquisition of radiology-specific competences in line with constructive alignment principles. Interdisciplinary case−based knowledge in the ICAT, by contrast, was assessed after 6 months because durable learning and knowledge transfer typically manifest after a consolidation phase. By separating immediate mastery from delayed application, the design aimed to minimize test contamination and to capture distinct learning outcomes targeted by the LP. The combination of synchronous interaction and asynchronous access corresponds to evolving learner expectations for hybrid, customizable formats [19]. The ability to revisit discussions, catch up on missed sessions, and selectively review content increases long-term educational use and accommodates diverse learning needs. These findings are consistent with prior institutional needs assessments showing strong preferences for flexible, multimodal tools [8].

### Didactic Implications for Teaching Practice

LPs are a cost-effective, low-threshold format that integrates readily into blended learning environments. The synchronous, case-based design suits the development of clinical reasoning and interdisciplinary thinking. Opportunities for learner questions and moderated dialogue promote interaction, reflection, and active engagement. High ratings in structure, relevance, and perceived benefit are known drivers of acceptance in higher education [20]. Embedding LPs as curriculum-aligned supplements, supported by preparatory and follow-up materials, can deepen learning and support knowledge retention. LPs are intended to complement, not replace, lectures so that students benefit from interactive digital formats while still using the lecture hall for consolidation and further integration of knowledge. Because LPs rely on existing audiovisual infrastructures and can be easily streamed, recorded, and integrated into learning management systems, this teaching format is scalable and transferable to other health professions programs.

### Limitations

The sample was small and restricted to a single center, which limits generalizability. Group allocation used consecutive cohorts rather than randomization, which may introduce selection bias. No pretest of baseline knowledge was conducted, and individual prior knowledge could not be controlled. The ICAT comprised 15 multiple-choice items and assessed midterm retention only, so effects on long-term learning and clinical performance remain unclear. The evaluation of student-reported acceptability in the SEQ relied on self-report, which may be affected by social desirability.

### Future Directions

Future research should use randomized, adequately powered, multicenter designs with baseline testing and correction for multiple comparisons. A longer follow-up is needed to examine durability. Performance-based outcomes such as objective structured clinical examinations and workplace-based assessments should be included to evaluate transfer to clinical decision-making and patient care. Studies could also compare LP design variants, for example, the degree of interactivity, scaffolding materials, and image integration.

### Conclusions

The LP was feasible and well accepted in the undergraduate DMFR course. It was associated with a significant knowledge gain in restorative dentistry and yielded results comparable to lectures in radiology-specific competences, while students rated it as more engaging and motivating. LPs, therefore, represent a scalable complement to standard instruction with potential to strengthen clinical reasoning and student-centered learning.

## Supplementary material

10.2196/77980 Multimedia Appendix 1 Live podcast episode 2—endodontology (case-based teaching material) PDF of the case-based teaching slides. The material includes learning objectives, a trauma-related case (teeth 21/22), sequential clinical and radiographic images (periapical radiographs [PA] and cone-beam computed tomography [CBCT]), key reasoning steps for pain diagnostics and pulp vitality assessment, take-home messages, and an optional self-test multiple-choice question (MCQ). All radiographs and clinical photographs are shown in anonymized form with separate patient consent for publication.

10.2196/77980 Multimedia Appendix 2 Interdisciplinary clinical application test (ICAT).

10.2196/77980 Multimedia Appendix 3 Radiology knowledge test (RKT).

10.2196/77980 Multimedia Appendix 4 Student Evaluation Questionnaire (SEQ).

## Abbreviations

CG: control group
CR: clinical reasoning
DMFR: dentomaxillofacial radiology
ICAT: interdisciplinary clinical application test
IG: intervention group
LP: live podcast
RKT: radiology knowledge test
SEQ: Student Evaluation Questionnaire
