# Supplementary material for: Live Podcasting as an Educational Intervention in Dentomaxillofacial Radiology: Controlled Cohort Study
Source: JMIR Med Educ. 2026 Jan 5;12:e77980. doi: 10.2196/77980 (PMC12768393; doi:10.2196/77980)
Supplement: Multimedia Appendix 1 [file mededu-v12-e77980-s001.pdf]

UNIVERSITÄTS KLINIKUM FREIBURG

Live Podcast
Dentomaxillofacial Radiology

Endodontology

Learning Objectives

- **Strengthen case-based reasoning:** Apply case-based clinical reasoning to diagnose dental pain and assess pulp vitality in order to make evidence-based treatment decisions.

- **Interpret findings in irreversible pulpitis and differentiate periapical pathologies:** Interpret clinical and radiographic findings associated with reversible and irreversible pulpitis and differentiate periapical pathologies; determine when CBCT is indicated in complex endodontic cases.

Case

- 29-year-old female patient (no significant medical history)

- Status: post bicycle accident with trauma to the upper jaw prior to initial presentation

- Initial management was provided by the *university emergency department* and the patient's general dentist:
  - Initial diagnosis: alveolar process fracture in region 21–22; lateral luxation of teeth 21 and 22
  - Initial therapy: splinting for 4 weeks
  - Close-interval monitoring of post-traumatic situation of teeth 21 and 22 by the patient's general dentist
  - Onset of spontaneous pain and fistula formation in region 21 → Referral to the *Center of Dental Medicine*

Clinical steps

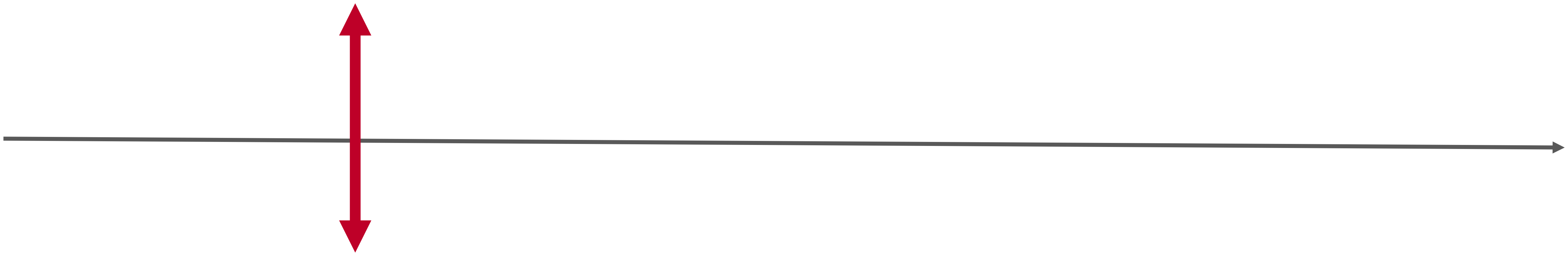

Radiological steps

Clinical and radiological steps

Clinical steps

Clinical signs:
21: non-vital, sensitive to vertical percussion testing
22: immediately positive cold response, not sensitive to vertical percussion testing

Therapy:
21: Start Root canal treatment
22: monitoring post traumatic situation

Radiological steps

Radiographs:
Periapical radiograph (PA):
Indication:
Follow-up trauma + Initial assessment RCT

Imaging modalities: PA

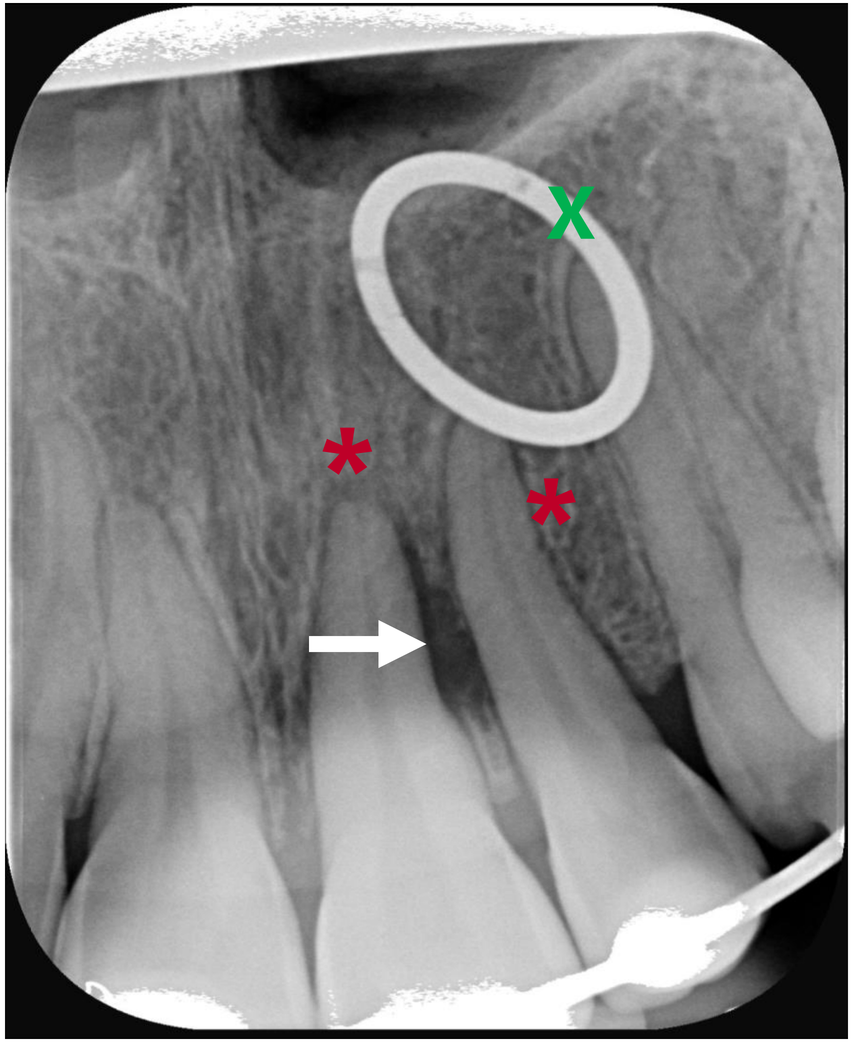

PA of teeth 21 and 22 at the patient's initial presentation.

Radiographic findings

- x *nasal piercing*
- * *widened periodontal ligament space (21 and 22)*
- ⇨ *interradicular radiolucency*
- *splint in situ*

! Unremoved objects such as jewelry may obscure important radiological findings

Clinical and radiological steps

Clinical steps

Clinical signs:
21: non-vital, sensitive to vertical percussion testing, fistula
22: immediately positive cold response, not sensitive to vertical percussion testing

Therapy:
21: Start Root canal treatment (RCT)
22: monitoring post traumatic situation

Clinical signs:
21: fistula, less pain
22: : non-vital, increasing pain, sensitive to vertical percussion testing

Therapy:
22: Start RCT

Clinical signs:
21 and 22: increased periodontal probing depths
After removing the splint: pronounced mobility of 21 and 22

Therapy:
Removal of splint (four weeks after trauma), Re-Application of splint, continuation of RCT

Radiological steps

Radiographs:
PA:
Indication:
Follow-up trauma + Initial assessment RCT

Radiographs:
Cone Beam-CT (CBCT):
Indication:
Further evaluation of suspected root fracture or root resorption in 21/22, insufficient diagnostic capability in 2D-imaging

Photograph:
Clinical situation

UNIVERSITÄTS KLINIKUM FREIBURG

Imaging modalities: CBCT

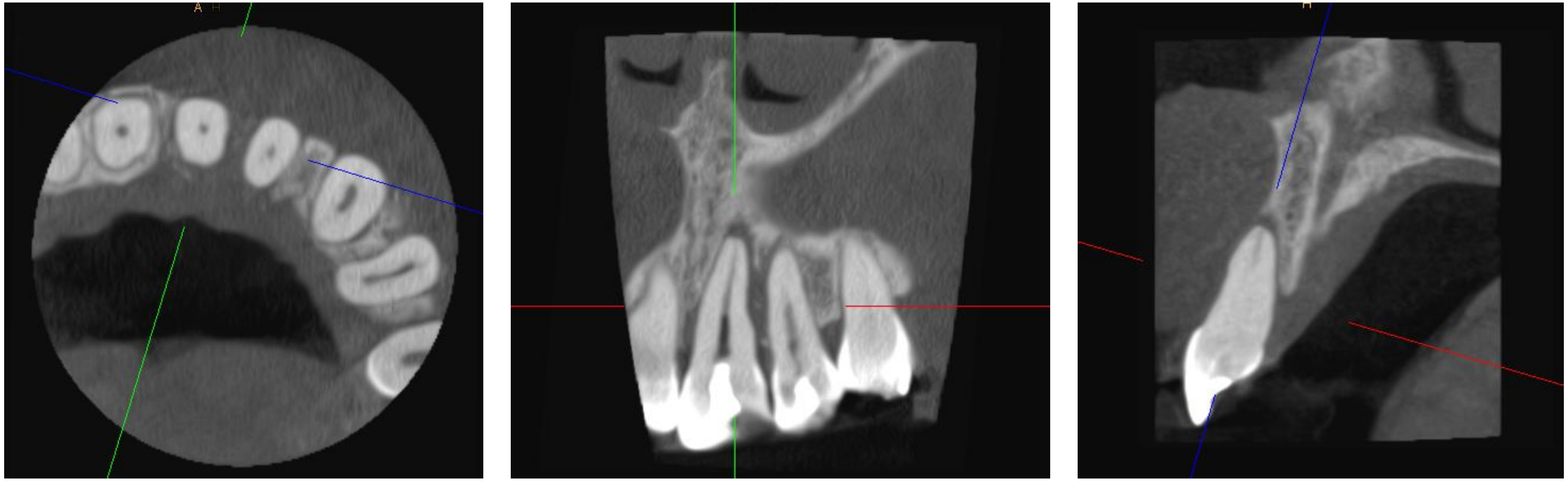

Axial, sagittal, and coronal section from the cone-beam computed tomography taken to rule out possible root fractures or root resorptions in 21 and 22.

<u>Radiographic findings</u>

- *widened periodontal ligament space 21 and 22*
- *no tooth fracture signs*
- *no tooth resorption signs*
- *Buccal bone not visible, palatinal and interradicular radiolucencies as signs of bone loss*

! CBCT only when 2D radiographs fail to provide diagnostic clarity, smallest feasible field of view, small voxel size, artifact-related masking of vertical root fractures is possible

Imaging modalities: CBCT

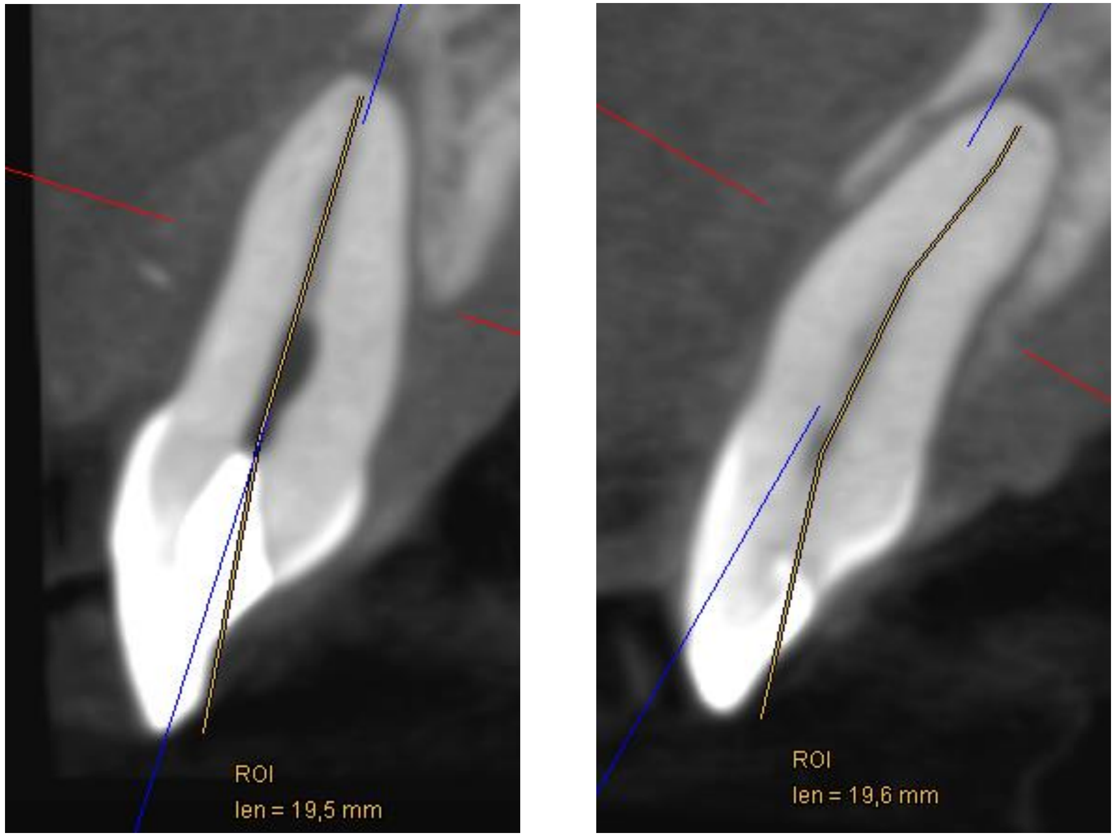

Screenshots from the cone-beam computed tomography with tooth 21 (left) and tooth 22 (right) to verify the measured working length of the canals.

! When there is a justifying indication for CBCT in this step, it may be utilized for working length determination

Clinical Photograph

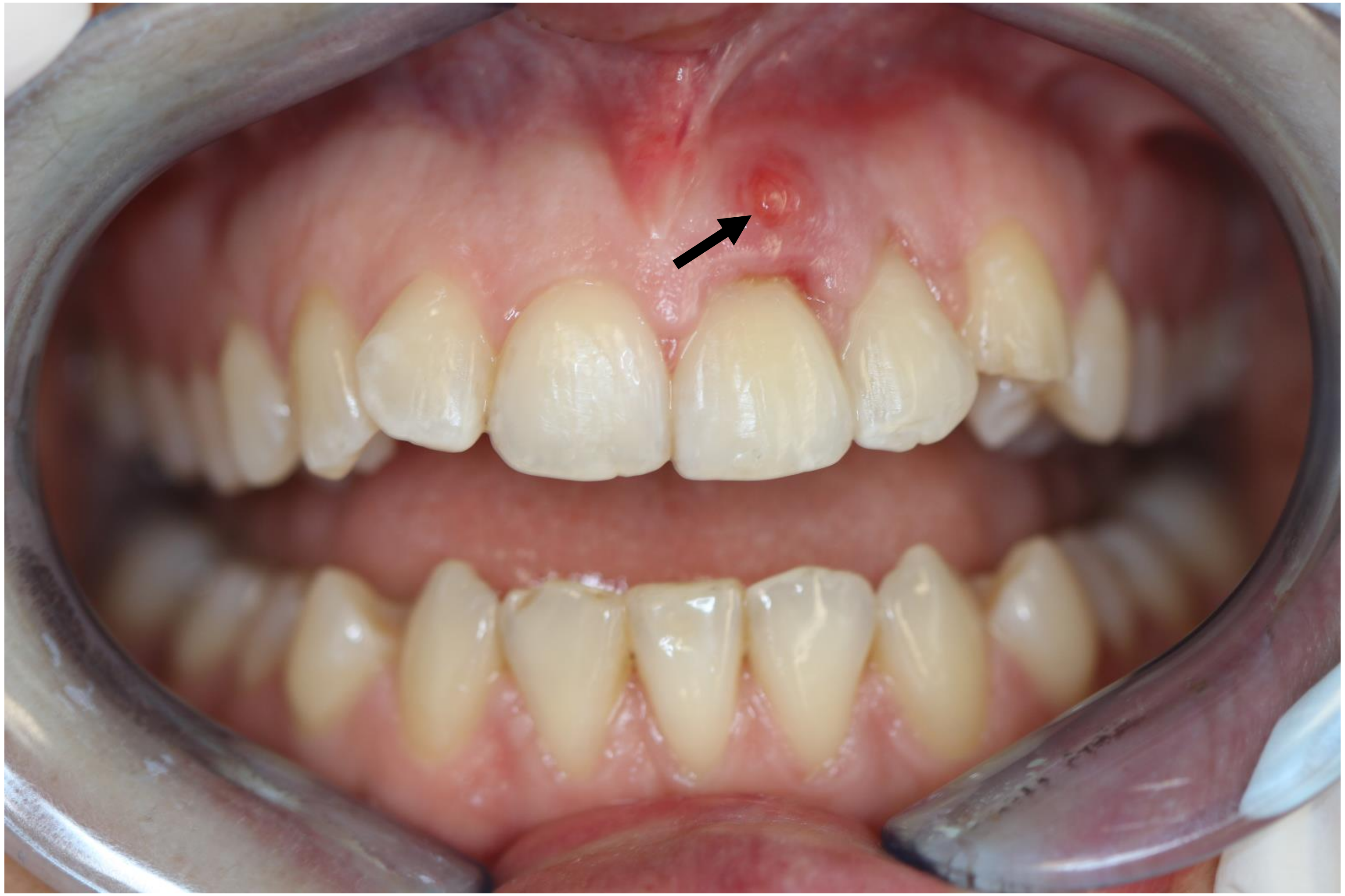

Clinical photograph after removal of the splint showing a buccal fistula in region 21 (→).

Clinical and radiological steps

Clinical steps

Clinical signs:
21: non-vital, sensitive to vertical percussion testing, fistula
22: immediately positive cold response, not sensitive to vertical percussion testing

Therapy:
21: Start Root canal treatment (RCT)
22: monitoring post traumatic situation

Clinical signs:
21: fistula, less pain
22: : non-vital, increasing pain, sensitive to vertical percussion testing

Therapy:
22: Start RCT

Clinical signs:
21 and 22: increased periodontal probing depths
After removing the splint: pronounced mobility of 21 and 22

Therapy:
Removal of splint (four weeks after trauma), Re-Application of splint, Continuation of RCT

Clinical signs:
21: no pain, fistula resolved
22: no pain

Therapy:
Continuation of RCT

Clinical signs:
21: no pain
22: no pain
both: reminescence of probing depths to physiological level

Therapy:
Continuation of RCT, Removal of splint (eight weeks after trauma), Root Canal Obturation

Radiological steps

Radiographs:
PA:
Indication:
Follow-up trauma + Initial assessment RCT

Radiographs:
Cone Beam-CT (CBCT):
Indication:
Further evaluation of suspected root fracture or root resorption in 21/22, insufficient diagnostic capability in 2D-imaging

Photograph:
Clinical situation

Radiographs:
PA:
Indication:
1. Confirmation of Working length and quality control prior to root canal obturation
2. Assessment of root canal obturation

UNIVERSITÄTS KLINIKUM FREIBURG

Imaging modalities: PA

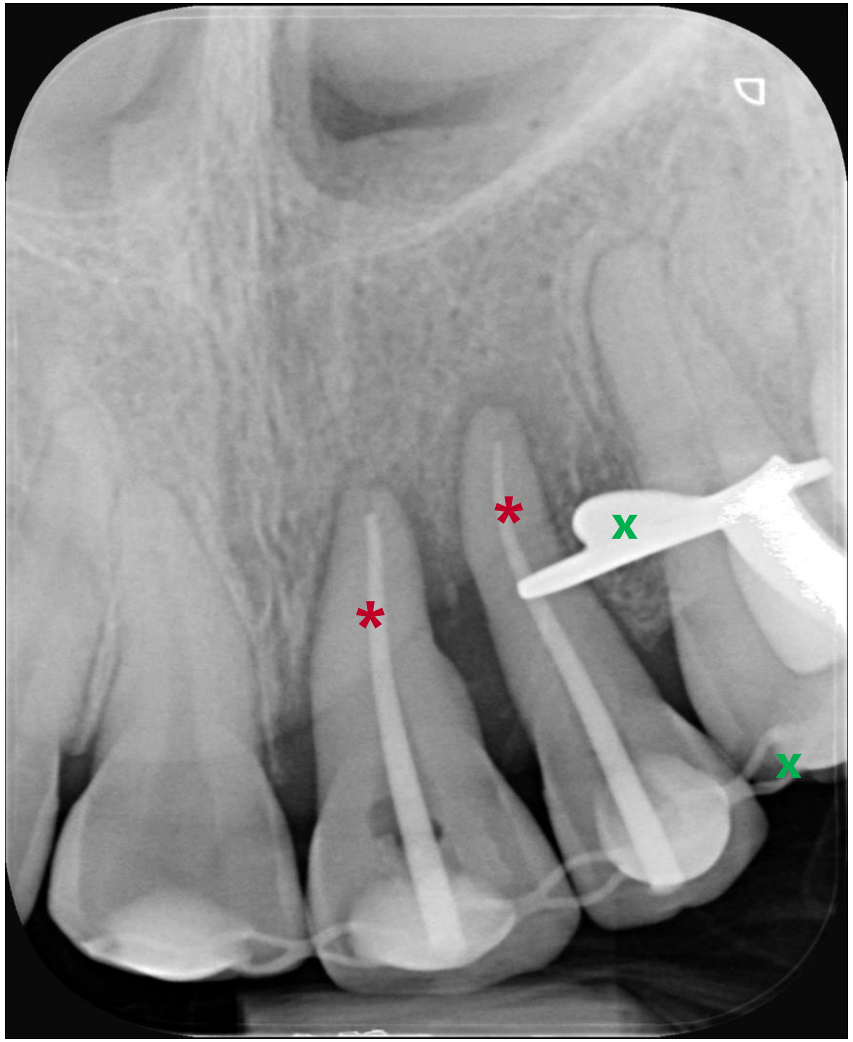

PA of teeth 21 and 22 serving as a control x-ray for the master apical gutta-percha points.

Radiographic findings

- *widened periodontal ligament space 21 and 22*
- *interradicular radiolucency*
- * *Master apical gutta-percha point with sufficient length and width*
- x *Rubber dam clamp and splint in situ*

UNIVERSITÄTS KLINIKUM FREIBURG

Imaging modalities: PA

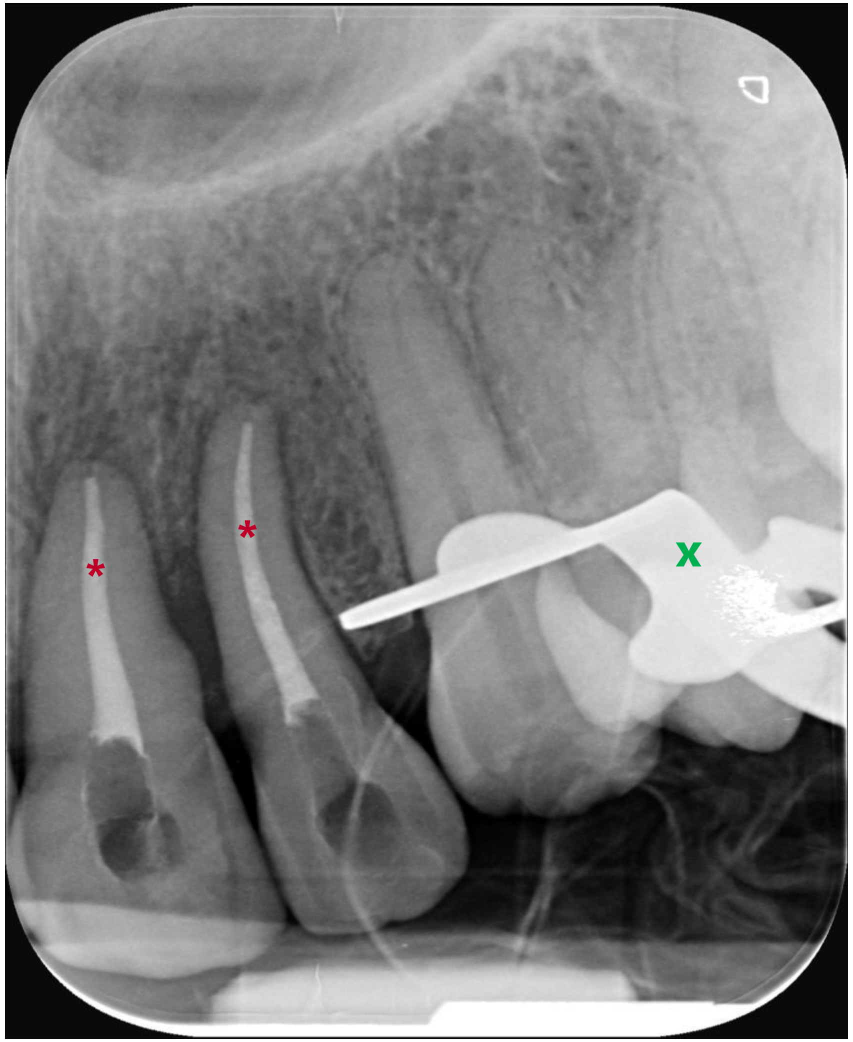

Periapical radiograph of teeth 21 and 22, serving as a follow-up image after root canal obturation.

Radiographic findings

- *widened periodontal ligament space 21 and 22*
- *interradicular radiolucency*
- * *Root Canal Obturation 21 and 22*
- x *Rubber dam clamp in situ*

Prognosis and Recall according to Dental Trauma Guide

- Risk of tooth loss

up to 1 year	up to 5 years	up to 10 years
less than 5 %	approx. 12 %	approx. 50 %

- Risk of infection-related resorption up to 10 years: less than 5 %

- Risk of marginal bone loss up to 10 years: less than 10 %

- Clinical and radiographic follow-up after
  - 8 weeks
  - 12 weeks
  - 6 months
  - 1 year
  - yearly for 5 years

! Follow-up mandatory, radiographs dependent on justifying indication

Key Reasoning Steps

Identify	Interpret	Prioritize	Integrate	Plan
– Specific medical history<br>– Pain characteristics<br>– Trigger<br>– Pulp vitality results<br>– PA findings (periapical lucency, PDL widening)	Radiographic and clinical correlation consistent with pulp status; exclusion of differential diagnoses.	Distinguish between conditions requiring immediate intervention vs. further diagnostics; determine urgency of treatment.	Combine clinical finding with findings of conventional PA to justify the need for CBCT based on the complexity of the case.	Define treatment approach, communicate risks and prognosis.

Take-Home Messages

- Diagnosis of trauma-related pulp injuries requires a combination of pulp vitality testing, clinical symptom assessment, and radiographic interpretation
- Periapical radiolucencies must be evaluated with regard to their etiology
- CBCT should only be performed when clearly indicated (e.g., complex anatomy, unclear findings)
- Radiographic findings should always be evaluated in conjunction with the clinical context
- Therapeutic decisions and prognosis are shaped by interdisciplinary considerations and following guidelines

Supplement- Optional Self-Test Item (MCQ)

Which statement is most applicable in the presented case?

**A**	A normal pulp vitality reliably rules out any kind of pulpitis
**B**	A periapical radiolucency may indicate an advanced apical periodontitis
**C**	Prolonged response to cold stimuli paired with periapical radiolucency indicates always irreversible pulpitis
**D**	CBCT is the first-line diagnostic tool in endodontics

Key Resources

- NKLZ: Competences Z5 („Zahnärztliche/r Experte/in“) and Z21 (Röntgendiagnostik, Strahlenschutz)
- DGZMK (2022). S2k-Leitlinie Therapie des dentalen Traumas bleibender Zähne. AWMF-Registernummer: 083-004
- Dental Trauma Guide: https://dentaltraumaguide.org
- Bourguignon C, Cohenca N, Lauridsen E, Flores MT, O'Connell AC, Day PF, Tsilingaridis G, Abbott PV, Fouad AF, Hicks L, Andreasen JO, Cehreli ZC, Harlamb S, Kahler B, Oginni A, Semper M, Levin L. International Association of Dental Traumatology guidelines for the management of traumatic dental injuries: 1. Fractures and luxations. Dent Traumatol. 2020 Aug;36(4):314-330. doi: 10.1111/edt.12578. Epub 2020 Jul 17. PMID: 32475015.
- European Society of Endodontology position statement: Use of cone beam computed tomography in Endodontics
- Tchorz JP, Wolgin M, Karygianni L, Vach K, Altenburger MJ. Accuracy of CBCT-based root canal length predetermination using new endodontic planning software compared to measurements performed with an electronic apex locator ex vivo. Int J Comput Dent. 2018;21(4):323-328. PMID: 30539174.
- DGZMK (2023). S2k-Leitlinie Dentale digitale Volumentomographie. AWMF-Registernummer: 083-005
- Clinical practice guidelines for endodontic treatment of the European Society of Endodontology (2023)
