# Supplementary material for: Live Podcasting as an Educational Intervention in Dentomaxillofacial Radiology: Controlled Cohort Study
Source: JMIR Med Educ. 2026 Jan 5;12:e77980. doi: 10.2196/77980 (PMC12768393; doi:10.2196/77980)
Supplement: Multimedia Appendix 2 [file mededu-v12-e77980-s002.pdf]

Test: Introducing Live-Podcasting in dental education: Designing and evaluating an interactive format for case-based and interdisciplinary learning

Question 1: Restorative Dentistry

A 45-year-old patient who is under your regular care comes to your practice without an appointment and reports pain in the left upper jaw that has persisted for several weeks. Tooth 26 has a mod composite filling and responds positively to percussion test and negatively to the cold test.

1. Your suspected diagnosis is irreversible pulpitis. To confirm your diagnosis, you take a bitewing x-ray.
2. The last panoramic x-ray (OPG) is more than six months old. To compare the size of the maxillary sinuses and rule out other pathologies, a new OPG should be taken.
3. You are unsure of your diagnosis and take an intraoral image.
4. The clinical symptoms are clear; the tooth is not worth saving. Following the ALARA principle, you do not take any x-rays to avoid unnecessary radiation exposition of the patient. You explain the extraction to the patient and extract the tooth.
5. Since dental CBCT is the imaging procedure with the highest resolution in dentistry and you want to assess the bone structures, you take a dental CBCT.

O All statements are incorrect.

O Only statement 1 is correct.

O The statements 1,2 and 3 are correct.

O Only statement 3 is correct.

O All statements are correct.

Question 2: Oral Surgery

Eight-year-old Max comes to your practice alone after falling off his bike. He reports pain in his jaw area. To rule out possible fractures and check for foreign objects, you decide to take x-rays. What do you need to bear in mind?

1. Since this is an emergency and you cannot reach his parents, you do not have to wait for their consent for the x-ray in order not to lose valuable time.
2. In the panoramic x-ray (OPG), you diagnose various approximal caries and recommend filling therapy for Max.
3. In the panoramic x-ray (OPG), you diagnose several fractures in the lower jaw and immediately refer Max to an oral surgeon.

O All statements are incorrect.

O Only statement 3 is correct.

O The statements 1 and 2 are correct

O All statements are correct.

O Only statement 1 is correct.

Question 3: Orthodontics

A 14-year-old female patient visits your general dental practice for the first time. During the clinical examination, you determine that the patient has a deep bite, missing teeth, and crowding of the lower front teeth. You decide to take X-rays to assess the situation more accurately and develop a treatment plan. Which of the following procedures is most appropriate?

1. Take a panoramic x-ray (OPG) to assess the tooth structure, root growth, and to ensure that there are no impacted teeth.
2. Take individual radiographs of the lower front teeth to document the crowding more accurately.
3. Do not take X-rays and instead take impressions of both jaws, as the situation models provide sufficient information.
4. Referral to an orthodontist.
5. Take a 3D image (Dental CBCT) with a large field of view as a basis for diagnosis.

O All statements are incorrect.

O The statements 2 and 4 are correct.

O Only statement 5 is correct.

O The statements 1 and 4 are correct.

O All statements are correct.
