# Supplementary material for: Live Podcasting as an Educational Intervention in Dentomaxillofacial Radiology: Controlled Cohort Study
Source: JMIR Med Educ. 2026 Jan 5;12:e77980. doi: 10.2196/77980 (PMC12768393; doi:10.2196/77980)
Supplement: Multimedia Appendix 3 [file mededu-v12-e77980-s003.pdf]

1. **Please choose the correct inspection intervals.**

	daily	weekly	monthly	annually	every 5 years
Resolution of line pairs					
Image receptor dose					
artifact-free imaging plates					
X-ray darkroom					

2. **Which of the following statements regarding radiobiological effects is correct?**

- ☐ A deterministic radiation effect occurs only after a defined threshold dose has been exceeded.
- ☐ A stochastic radiation effect occurs only after a defined threshold dose has been exceeded.
- ☐ Damage to gametes is classified as a deterministic radiation effect.

3. **Which incidental finding in panoramic radiography often appears as multiple small round opacities projected onto the mandibular angle?**

- ☐ Projection of tonsilliths
- ☐ Projection of tonsolliths
- ☐ Projection of tonsollililiths
- ☐ Projection of tonsilloliths

4. Which of the following statements regarding the panoramic radiograph (OPG) is correct?

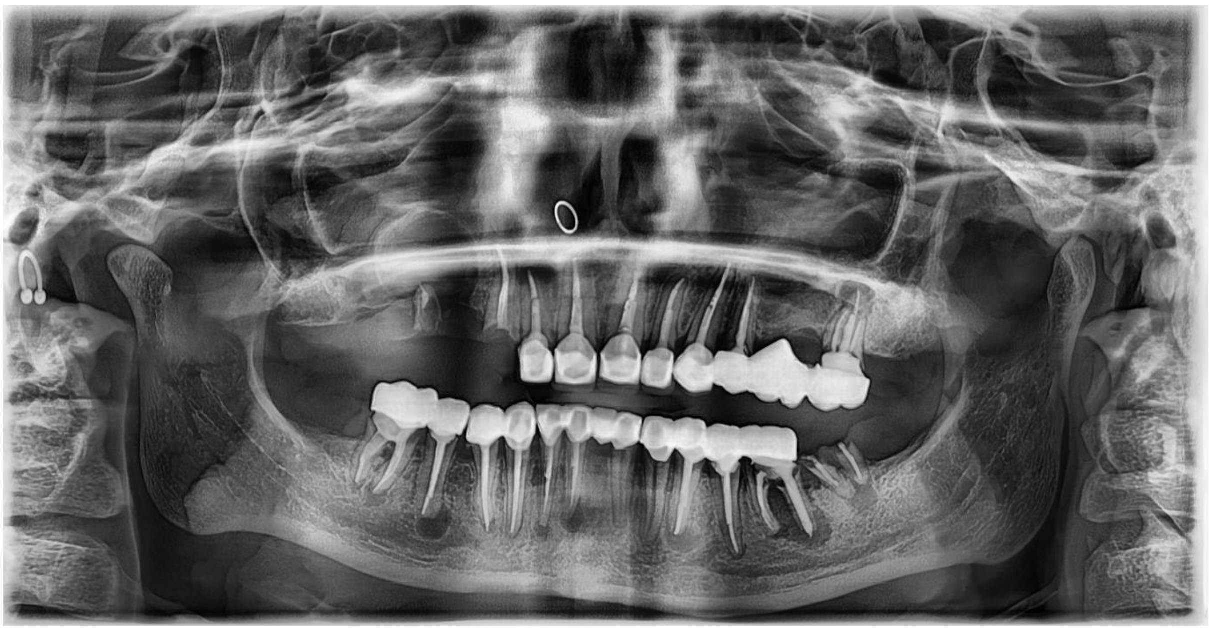

- ☐ The circular, well-defined radiolucency in region 15 raises suspicion of a Stafne cyst.
- ☐ The metal-dense, ring-shaped opacity in the apical region of tooth 11 is consistent with a palatal implant.
- ☐ The coronal opacities in regions 36–46 are consistent with promethic and restorative work.
- ☐ The intraradicular opacities in regions 15, 13, 12, 11, 21, 22, 23, 24, 27, 37, 36, 35, 34, 41, 42, 43, 44, 45, 46 are consistent with root canal fillings.
- ☐ A well-defined linear radiolucency at the right mandibular angle is compatible with a non-displaced fracture of the right mandibular angle.

5. Please mark the statement that is not correct regarding intraoral radiographs.

- ☐ In the paralleling technique, the central ray strikes the film plane at a right angle.
- ☐ In the paralleling technique, the central ray strikes the long axis of the tooth at a right angle.
- ☐ In the right-angle (bisecting) technique, the central ray strikes the film plane at a right angle.
- ☐ In the right-angle (bisecting) technique, the central ray strikes the long axis of the tooth at a right angle.

6. **Which of the following requirements for operating a type-approved X-ray unit is not mandatory?**
   - ☐ The certificate of expertise must be submitted to the competent authority.
   - ☐ The expert inspection report must be submitted to the competent authority.
   - ☐ The approved application must be available.
   - ☐ The commissioning must be reported to the competent authority in a timely manner.

7. **How often must radiation protection certification be renewed?**
   - ☐ Annually
   - ☐ Every 2 years
   - ☐ Every 5 years
   - ☐ Every 7 years

8. **Which statement regarding the assessment of an X-ray image is not correct?**
   - ☐ All findings should be described in terms of location, shape, margins, internal structure, density, and their relationship to surrounding structures.
   - ☐ The term “opacity” refers to areas that appear darker, while “radiolucency” refers to areas that appear lighter.
   - ☐ Distortion occurs whenever the object is not positioned parallel to the detector plane.

9. **Using parallax shift, it is possible to determine whether objects are located buccally or orally (lingually/palatally) relative to the dental arch. Please mark the correct position of the paperclip according to the principle of parallax shift.**

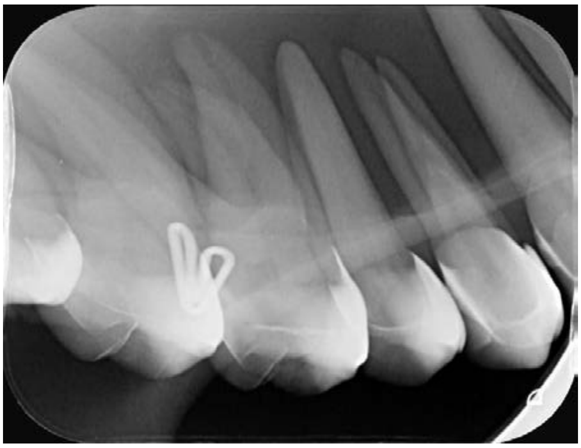

mesioangular projection

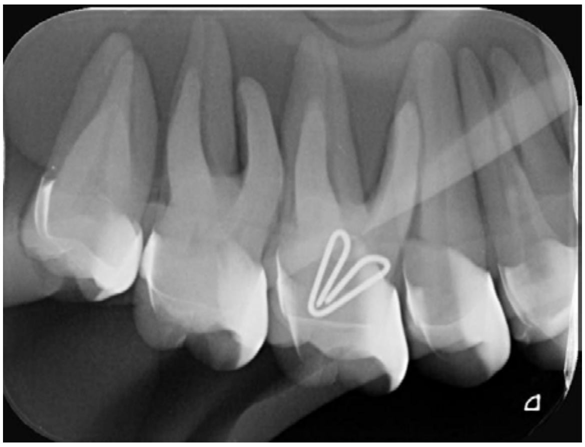

distoangular projection

   - ☐ The paperclip is located buccal to the dental arch.
   - ☐ The paperclip is located palatal to the dental arch.

10. If you were to use a storage phosphor plate labeled with a permanent marker on a patient, the labeling would appear on the screen as follows:

- ☐ white: e.g. 1
- ☐ black: e.g. 1
- ☐ not visible

11. What radiation protection certification is required for a dentist who does not take CBCT scans themselves but interprets the findings?

- ☐ No special certification
- ☐ Medical radiation protection certification
- ☐ Dental CBCT (cone-beam CT) radiation protection certification

12. In the bisecting-angle technique, a tooth appears shortened. What positioning error has occurred?

- ☐ A distal shift radiograph was accidentally taken
- ☐ The angulation was too steep
- ☐ The angulation was too flat
- ☐ The angulation was correct

13. Which differential diagnosis typically appears radiographically as a poorly defined radiolucency in the area of tooth apex?

- ☐ Acute lateral periodontitis
- ☐ Chronic lateral periodontitis
- ☐ Acute apical periodontitis
- ☐ Chronic apical periodontitis

14. Which statement regarding patient positioning in panoramic radiography (OPG) is incorrect?

- ☐ If the head is rotated to the left while the midline is aligned, the left molars appear widened
- ☐ If the patient is positioned too far posteriorly in the unit, the teeth appear widened
- ☐ The “smiling patient” occurs when the head is tilted too far backward
- ☐ In the “sad patient,” the ascending mandibular rami diverge

15. Which statement regarding the radiation protection area is correct?

- ☐ The natural radiation exposure in Germany is at least 1 mSv/h.
- ☐ The effective dose within the controlled area is more than 6 mSv/h.
- ☐ The dose rate within the restricted area is at least 3 mSv/h.
- ☐ The effective dose within the supervised area is more than 1 mSv/h.

16. In which clinical scenario is the use of cone-beam computed tomography (CBCT) not indicated?

- ☐ Assessment of vertical and horizontal bone volume for implant planning
- ☐ Diagnosis of maxillary sinus diseases
- ☐ Temporomandibular joint imaging to evaluate soft tissues such as the articular disc
- ☐ Advanced endodontic diagnostics when basic imaging is insufficient

17. How long must radiographs of an 11-year-old patient be retained?

- ☐ Not at all, radiographs are always given to the patient for safekeeping
- ☐ 10 years
- ☐ 5 years
- ☐ Until the patient reaches the age of 28

18. Name a radiological differential diagnosis for “chronic apical periodontitis” in a carious tooth 36 of a 7-year-old patient.

- ☐ Cementoma
- ☐ Incomplete root development
- ☐ Juvenile xanthogranuloma
- ☐ Histiocytoma

19. Which novel anode concepts are you familiar with?

- ☐ Cathoden-anode
- ☐ Liquid anode
- ☐ Gaseous anode
- ☐ Synode

20. Which of the following is not a component of a conventional X-ray film?

- ☐ Adhesive layer
- ☐ Protective layer
- ☐ Varnish layer
- ☐ Base layer

21. Which reconstruction method allows for trajectories that deviate from the rotation of the detector?

- ☐ Filtered back projection
- ☐ Parallel-series feedback
- ☐ Iterative image reconstruction
