# Supplementary material for: Live Podcasting as an Educational Intervention in Dentomaxillofacial Radiology: Controlled Cohort Study
Source: JMIR Med Educ. 2026 Jan 5;12:e77980. doi: 10.2196/77980 (PMC12768393; doi:10.2196/77980)
Supplement: Multimedia Appendix 4 [file mededu-v12-e77980-s004.pdf]

Event framework

Attendance

I attended the Live-podcast.

O yes

O no

Gender

I am...

O a female

O a male

O I dont want to specify

Organisation

I was satisfied with the organization prior to the Live-Podcast.

O	O	O	O	O	O	O	O	O	O
1(I strongly disagree)	2	3	4	5	6	7	8	9	10(I strongly agree)

Lecture hall and equipment

I was satisfied with the selection of the lecture hall and equipment.

O	O	O	O	O	O	O	O	O	O
1(I strongly disagree)	2	3	4	5	6	7	8	9	10(I strongly agree)

Provision

I was satisfied with the online availability of the Live-Podcast episodes afterwards.

O	O	O	O	O	O	O	O	O	O
1(I strongly disagree)	2	3	4	5	6	7	8	9	10(I strongly agree)

Event course

Evaluation of the Length

I was satisfied with the length of the Live-Podcast.

O	O	O	O	O	O	O	O	O	O
1(I strongly disagree)	2	3	4	5	6	7	8	9	10(I strongly agree)

Atmosphere

I perceived the atmosphere of the Live-Podcast as pleasant.

O	O	O	O	O	O	O	O	O	O
1(I strongly disagree)	2	3	4	5	6	7	8	9	10(I strongly agree)

Interest in Topics

I was interested in the topics of the Live-Podcast.

O	O	O	O	O	O	O	O	O	O
1(I strongly disagree)	2	3	4	5	6	7	8	9	10(I strongly agree)

Contribution I

In comparison to standard lectures, I felt more confident to request to speak.

O	O	O	O	O	O	O	O	O	O
1(I strongly disagree)	2	3	4	5	6	7	8	9	10(I strongly agree)

Contribution II

As the semester progressed, I felt increasingly confident to request to speak.

O	O	O	O	O	O	O	O	O	O
1(I strongly disagree)	2	3	4	5	6	7	8	9	10(I strongly agree)

Level and demands

The demands on the audience were not too high.

O	O	O	O	O	O	O	O	O	O
1(I strongly disagree)	2	3	4	5	6	7	8	9	10(I strongly agree)

Online replay

After the Live-Podcast I replayed the episodes online.

O	O	O	O	O	O	O	O	O	O
1(I strongly disagree)	2	3	4	5	6	7	8	9	10(I strongly agree)

Learning success

Here you should compare your own learning success compared to lectures that don't use a Live-Podcast as an additional teaching method.

Because of the support of the standard lectures by the Live-Podcast...

Focus on content

I was able to concentrate better on the content in comparison to standard lectures.

O	O	O	O	O	O	O	O	O	O
1(I strongly disagree)	2	3	4	5	6	7	8	9	10(I strongly agree)

Distraction

I was less distracted in comparison to standard lectures.

O	O	O	O	O	O	O	O	O	O
1(I strongly disagree)	2	3	4	5	6	7	8	9	10(I strongly agree)

Memorization

I was able to memorize more in comparison to standard lectures.

O	O	O	O	O	O	O	O	O	O
1(I strongly disagree)	2	3	4	5	6	7	8	9	10(I strongly agree)

Long-term memory

I was able to retain the content better in comparison to standard lectures.

O	O	O	O	O	O	O	O	O	O
1(I strongly disagree)	2	3	4	5	6	7	8	9	10(I strongly agree)

Attentionspan

I was able to follow the content for a longer time in comparison to standard lectures.

O	O	O	O	O	O	O	O	O	O
1(I strongly disagree)	2	3	4	5	6	7	8	9	10(I strongly agree)

Comprehension

I was able to comprehend the content more easily in comparison to standard lectures.

O	O	O	O	O	O	O	O	O	O
1(I strongly disagree)	2	3	4	5	6	7	8	9	10(I strongly agree)

Scope of knowledge

I was able to expand my scope of knowledge more.

O	O	O	O	O	O	O	O	O	O
1(I strongly disagree)	2	3	4	5	6	7	8	9	10(I strongly agree)

Learning from the experiences of others

I was able to learn more from the experiences of the teachers and the students in comparison to standard lectures.

O	O	O	O	O	O	O	O	O	O
1(I strongly disagree)	2	3	4	5	6	7	8	9	10(I strongly agree)

Practical reference

I was able to connect theory and practice more in comparison to standard lectures.

O	O	O	O	O	O	O	O	O	O
1(I strongly disagree)	2	3	4	5	6	7	8	9	10(I strongly agree)

Interdisciplinary connections

I was better able to recognize and understand interdisciplinary connections.

O	O	O	O	O	O	O	O	O	O
1(I strongly disagree)	2	3	4	5	6	7	8	9	10(I strongly agree)

Multi-level therapeutic approach

I became more aware of multi-level therapeutic approaches (orthodontics-oral surgery-prosthetics-restorative dentistry-general medicine).

O	O	O	O	O	O	O	O	O	O
1(I strongly disagree)	2	3	4	5	6	7	8	9	10(I strongly agree)

Overall rating

Useful teaching method

I consider the Live-Podcast as an additional teaching method to be meaningful.

O	O	O	O	O	O	O	O	O	O
1(I strongly disagree)	2	3	4	5	6	7	8	9	10(I strongly agree)

Recommendation

I would recommend the Live-Podcast to students of earlier semesters.

O	O	O	O	O	O	O	O	O	O
1(I strongly disagree)	2	3	4	5	6	7	8	9	10(I strongly agree)

Encouraged reflection

The Live-Podcast was thought-provoking.

O	O	O	O	O	O	O	O	O	O
1(I strongly disagree)	2	3	4	5	6	7	8	9	10(I strongly agree)

Personal perception

Because of the Live-Podcast my interpretation and perception of the field of dentistry changed.

O	O	O	O	O	O	O	O	O	O
1(I strongly disagree)	2	3	4	5	6	7	8	9	10(I strongly agree)

Addition to the lecture

In addition to standard lectures, I would like to listen to a Live-Podcast format every semester.

O	O	O	O	O	O	O	O	O	O
1(I strongly disagree)	2	3	4	5	6	7	8	9	10(I strongly agree)

Feedback

Here you could give feedback or suggest improvements:
